# Molecular Genotyping of Circulating Enterovirus in the Lazio Region from 2012 to 2023

**DOI:** 10.3390/v16071013

**Published:** 2024-06-24

**Authors:** Martina Rueca, Francesco Vairo, Martina Spaziante, Lavinia Fabeni, Federica Forbici, Giulia Berno, Cesare Ernesto Maria Gruber, Simonetta Picone, Camilla Ajassa, Enrico Girardi, Fabrizio Maggi, Maria Beatrice Valli

**Affiliations:** 1 Laboratory of Virology, National Institute for Infectious Diseases “Lazzaro Spallanzani” IRCCS, 00149 Rome, Italy; martina.rueca@inmi.it (M.R.); federica.forbici@inmi.it (F.F.); giulia.berno@inmi.it (G.B.); cesare.gruber@inmi.it (C.E.M.G.); fabrizio.maggi@inmi.it (F.M.); mariabeatrice.valli@inmi.it (M.B.V.); 2 Regional Service for Surveillance and Control of Infectious Diseases (SERESMI)-Lazio Region, National Institute for Infectious Diseases “Lazzaro Spallanzani” IRCCS, 00149 Rome, Italy; francesco.vairo@inmi.it (F.V.); martina.spaziante@inmi.it (M.S.); 3 Neonatology and Neonatal Intensive Care Unit, Policlinico Casilino, 00169 Rome, Italy; simpico@libero.it; 4 Department of Public Health and Infectious Diseases, Sapienza University Hospital “Policlinico Umberto I”, 00161 Rome, Italy; camilla.ajassa@uniroma1.it; 5 Scientific Direction, National Institute for Infectious Diseases “Lazzaro Spallanzani” IRCCS, 00149 Rome, Italy; enrico.girardi@inmi.it

**Keywords:** enterovirus, sequencing, phylogenesis, molecular epidemiology, enterovirus typing

## Abstract

Enteroviruses (EVs) are ubiquitous viruses that circulate worldwide, causing sporadic or epidemic infections, typically during the summer and fall. They cause a broad spectrum of illnesses, ranging from an unspecified febrile clinical presentation to a severe illness. EVs are recognized to be the most frequent etiological agents of aseptic meningitis in children. However, as the infection is usually mild and self-limiting, it remains underestimated, and the epidemiology of EVs is poorly understood. To date, no vaccine or effective therapy for all types of enteroviruses is available, and EVs constitute a public health concern. Here, we investigated the molecular epidemiology of EV strains circulating in the Lazio region over a 10-year time span (2012–2023) by using a sequence-typing approach and phylogenetic analysis. The epidemiological trend of EV infection has undergone changes during the SARS-CoV-2 pandemic (2020–2021), which resulted in a modification in terms of the number of diagnosed cases and seasonality. From 2022, the circulation of EVs showed a behavior typical of the pre-pandemic period, although changes in predominantly circulating strains have been noted. Both epidemic and sporadic circulation events have been characterized in the Lazio region. Further analyses are needed to better characterize any strain with higher potential pathogenic power and to identify possible recombinant strains.

## 1. Introduction

Enteroviruses (EVs) are among the most common viruses worldwide and they are characterized by a great genotypic and phenotypic variability. In fact, they are responsible for a wide spectrum of clinical illness, ranging from mild respiratory or gastrointestinal symptoms to severe clinical features such as acute and chronic cardiac disease, hepatitis, meningitis and encephalitis. The transmission of the infection is generally sustained by the fecal–oral route, but may also occur via respiratory droplets [1,2]. EVs belong to a large genus of small icosahedral non-enveloped viruses with a single stranded positive-sense RNA genome and are included in the *Picornaviridae* family, which consists of 15 species including over 100 different types (formerly called “serotypes” due to identification by a cross-neutralization assay); among them, only seven (Enterovirus A to D and Rhinovirus A to C) have been found to infect humans [3]. Before the genetic interrelationships among the member of EVs were understood, the serotypes were assigned based on a biologically based classification system and designated as echovirus (abbreviation “E”), coxsackievirus A (abbreviation “CVA”) and coxsackievirus B (abbreviation “CVB”), where A and B do not refer to the species but historically depend on whether they caused flaccid or spastic paralysis. Today, the serological classification of subspecies has been replaced by a molecular method based on the nucleotide sequence of the hypervariable capsid VP1 gene [4,5,6]. As a consequence, ICTV recommended using the term “type” rather than “serotype” for nomenclature of all EVs [4], while all the newly identified ones are named numerically and designated as “EV” followed by the species letter. Given the rapid evolution of the genus, new types are continuously identified, such as EV-B80, EV-B83 and EV-B93, reported in China in the last few years [7,8,9].

The genome of EVs is a single stranded positive-sense RNA of about 7.5 kb in length that contains a single Open Reading Frame (ORF) encoding a unique polyprotein and flanked by two well-conserved and untranslated regions (UTR) at 5′ and 3′, respectively, that play an important functional role in the viral biology [10].

Due to the low-fidelity replication of the RNA-dependent RNA polymerase that lacks proofreading activity and a high frequency of recombination events, the genome of EVs evolves at a high mutation rate. According to previous studies, Poliovirus and Non-Polio Enterovirus’ (NP-EVs) VP1 gene accumulates 1.35 to 5 × 10^−3^ and 5 to 15 × 10^−3^ substitutions per site per year, respectively [11,12,13].

Over the past decades, some specific types that were previously known to be associated with sporadic and mild infections, such as Enterovirus A71 (EV-A71) and Enterovirus D68 (EV-D68) have emerged as serious public health threats. The EV-A71 was commonly implicated in Hand, Foot and Mouth Diseases (HFMD) since the late 1990s, when it caused large outbreaks in Asia associated with an increased risk of neurological disease [11,14]. While Enterovirus D68 (species D), known for years as a rare respiratory pathogen, caused a large outbreak in the United States with an apparent increase in acute flaccid paralysis (AFP) rates in 2014 [15], since then it has started to spread globally and cases are on the rise, as recently reported [15,16].

More recently, in April 2023, the National IHR Focal Point for the United Kingdom informed the WHO of an increase in severe myocarditis in neonates associated with enterovirus infection in Wales. Typing characterization revealed either coxsackie B3 or coxsackie B4. Seven cases were treated in intensive care, and one case died before transfer to tertiary care [17].

Since July 2022, neonates with severe sepsis leading to hepatic failure and neurological or myocardial involvement caused by infection with Echovirus 11 (E-11) have been diagnosed in France; seven of them died [18]. As a result, the Organization World Health Authority launched an alert that quickly led to the identification of other cases in Italy, Spain, Croatia and the United Kingdom [19,20,21]. 

The epidemiology of EVs in temperate regions of the world is characterized by constant circulation with epidemic peaks corresponding to the summer and fall seasons. However, in Italy, there is not a systematically organized surveillance program, so is data derived mainly through passive surveillance, and, therefore, a sufficiently reliable picture of EV circulation is unknown.

The use of a sequencing approach for typing of EVs cases, associated with phylogenetic analysis, has proven to be a reliable method for monitoring the circulation dynamics of different NP-EV types and a valid tool to rapidly identify the emergence of new EV variants and epidemic strains with possibly higher pathogenic power.

Here we report data on the diagnosis, molecular characterization and phylogenetic analysis of NP-EVs in the Lazio Region in the 10-year period spanning from 2012 to 2023 to get a picture of the circulation of such viruses and identify both endemic and epidemic circulating strains, evaluate transmission dynamics and identify clusters of infection.

## 2. Materials and Methods

### 2.1. Collection of Samples

Different types of specimens from patients with suspected EV infection were analyzed for the presence of EV RNA at the laboratory of Virology at the National Institute for Infectious Diseases “L. Spallanzani” (INMI), and those that tested positive were collected and retrospectively sequenced for typing purposes. 

The biological samples analyzed included: oropharyngeal swabs, rectal swabs, feces, cerebrospinal fluid (CSF), serum, plasma, broncho-alveolar lavage (BAL), sputum and tissue bioptic and autoptic samples.

### 2.2. Diagnosis and Typing

EV RNA was detected by commercial methods from collected samples of patients who underwent molecular diagnosis (One-Step qRT-PCR). The viral RNA was extracted from clinical samples using the Qiasymphony automatic extractor (QIAGEN), and RNA amplification of the partial VP1 and UTR gene sequencing was performed using the Qiagen OneStep RT-PCR kit (QIAGEN) according to manufacturer’s instructions. The 5′UTR partial region was amplified using primers described by Nicholson et al. [21] to obtain species characterization, while amplification of a portion of the VP1 gene was obtained using primers AN88–AN89, by Nix et al. or species-specific home-made primers [22], according to viral load. Primers sequences are provided in the Supplementary Materials (File S1).

The amplicons of 5′UTR and VP1 were sequenced using the ABI prism 3130x1 GENETIC ANALYZER DNA Sequencer. The EV species and genotype identification were determined by analyzing the nucleotide sequences of partial lengths of the 5′UTR and VP1, respectively, with the web-based, open-access EV Genotyping Tool Version 0.1 (https://www.rivm.nl/mpf/typingtool/enterovirus/, accessed on 1 April 2024) [23]. Species assignment was obtained based on the partial sequence of 5′UTR, while the VP1 partial sequence was used for serotype assignment. 

### 2.3. Phylogenetic Analysis

Two phylogenetic analyses were performed comparing the VP1 partial sequences INMI datasets and the 5′UTR partial sequences INMI datasets (70 and 90 sequences for the VP1 and 5′UTR regions, respectively) with 112 prototype reference sequences retrieved from NCBI, whose accession numbers were selected from the Picornaviridae homepage databases (File S2) [3]. The INMI sequences suitable for phylogeny were chosen on the basis of the quality and length of base pairs obtained. In detail, for 5UTR, sequences with a minimum length of 125 nt were used, and for VP1 a minimum of 290. The VP1 and 5′UTR trees were constructed using the neighbor-joining (NJ) method [24], by MEGA 7 [25]. The reliability of the branching orders was assessed by a bootstrap analysis of 1000 replicates [26]. The general time-reversible (GTR) nucleotide substitution model with gamma distribution among site heterogeneity (G+I) was considered the best one by the MEGA 7 model test, as it showed the lowest BIC (Bayesian Information criterion) score. A sequence that clustered monophyletically inside a clade with a bootstrap support value of ≥80% was assigned to that clade. The trees were rooted using midpoint rooting by FigTree software version 1.4.4 [27].

## 3. Results

### 3.1. EV Diagnostic Activity over Ten Years 2012–2023: Samples Analyzed and Percentage of Positive Cases

We examined the data obtained from the EV diagnostic activity from January 2012 to December 2023. Over the course of ten years 3346 specimens collected from 2689 patients were tested for the presence of EV RNA; of them, 284 were found positive for EV RNA, corresponding to 184 patients (6.8% of total patients tested). The median age of patients was 25 years old (IQR 3–35.3), 55% male, 45% female.

**Figure 1:**
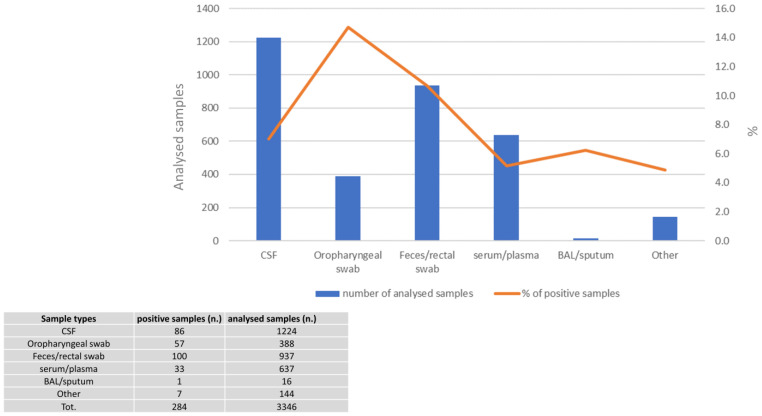
Sample types for EV diagnosis over the period 2012–2023.

**Figure 2:**
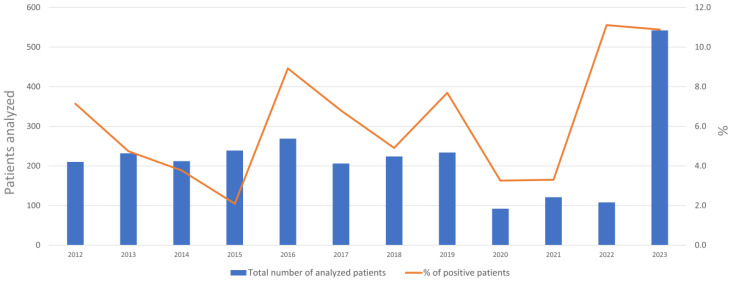
EV diagnosis over the period 2012–2023.

Analyzed specimens were mostly represented by CSF, followed by feces/stool and serum/plasma (blue bars in Figure 1), while major percentage of positivity (orange line in Figure 2) was observed for oropharyngeal swabs, followed by feces/stool and lastly CSF (14.7, 10.7 and 7%, respectively). 

The graph in Figure 2 reports the total number of patients who underwent laboratory molecular EV diagnosis during the study period (blue bars), distributed per year; positivity rates are also shown (orange line). The graph shows that the number of patients tested was almost constant (ranging from 206 to 269) before the three-year SARS-CoV-2 pandemic window (2020–2022), during which there was, as expected, a recorded drop of about 50%; on the other hand, a sharp increase in diagnoses was observed in 2023, with a total of 543 patients tested (Figure 2). As shown in the graph, the percentage of positivity did not parallel the number of patients tested, but revealed a year to year change in the trend of EV infections, with a huge increase of cases observed in the last two years (2022 and 2023). 

**Figure 3:**
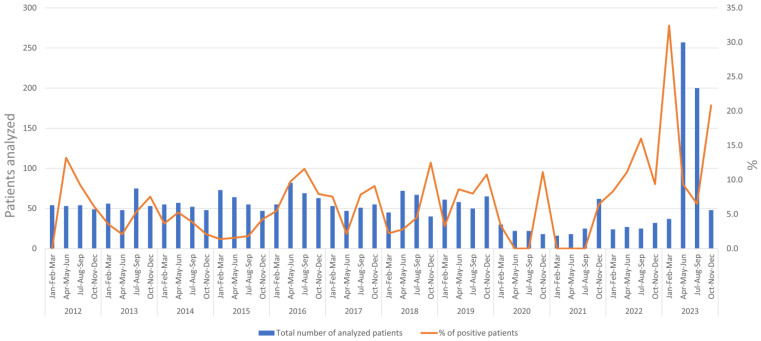
Seasonality of EV cases diagnosed at INMI over the period 2012–2023.

To investigate the seasonal trend of EV infections over the ten-year period examined, we analyzed the number of patients tested for EVs (blue bars) and the percentage of positive cases (orange line) distributed per trimester. Figure 3 shows that the number of EV infections in the Lazio region fluctuates, with peaks mostly observed in the summer and autumn months (June to December). Of note, the highest peak of cases was observed in 2023 (in particular from April to September). As shown in the figure, the percentage of EV positive cases increased from autumn 2022 with a major peak occurring in the autumn–winter period of 2022 and 2023 (October 2022 to March 2023) when 30% of analyzed patients tested positive for EV infection. 

### 3.2. Typing of EV Cases over the Period 2012–2023

**Figure 4:**
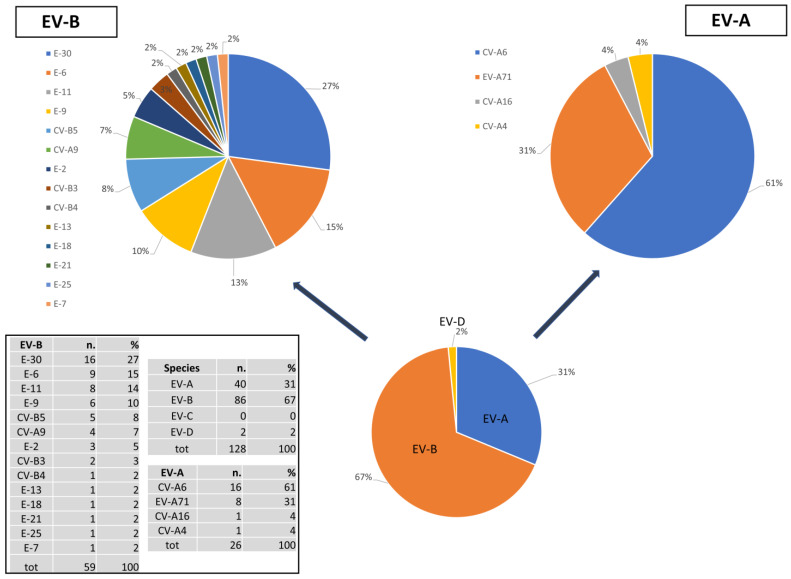
Molecular typing of EV strains circulating in the Lazio region (2012 to 2023).

To evaluate the EV species and types circulating in the Lazio region over the ten-year period studied, 128 of 184 positive cases could be typed for species assignment, corresponding to 69.5% of cases; while only of 85 cases could be sequenced to assign the serotype based on VP1 region (46.7% of total cases). As far as concerns species assignment obtained on the basis of the Enterovirus Genotyping Tool, EV-B species (67%) is the most common, followed by EV-A species (31%) and EV-D species (2%); cases of infection caused by EV of C species, to which belong the poliovirus types, were not found (Figure 4). However, the species frequencies might not be accurate due to those strains of which we had only 5UTR partial sequences for the eventual presence of recombinant strains.

Overall, we detected 14 different EV-B types, among which the most frequent were E-30, E-6 and E-11 (27%, 15%, and 14% of B species cases, respectively). With regard to A species, the most represented types were CV-A6 followed by EV-A71 (61% and 31%, respectively). Of interest, concerning the EV-D species, one case due to EV-D68 was identified in a CSF sample [28].

### 3.3. Phylogenetic Analysis of EV Circulating in the Lazio Region over the Period 2012–2023

**Figure 5:**
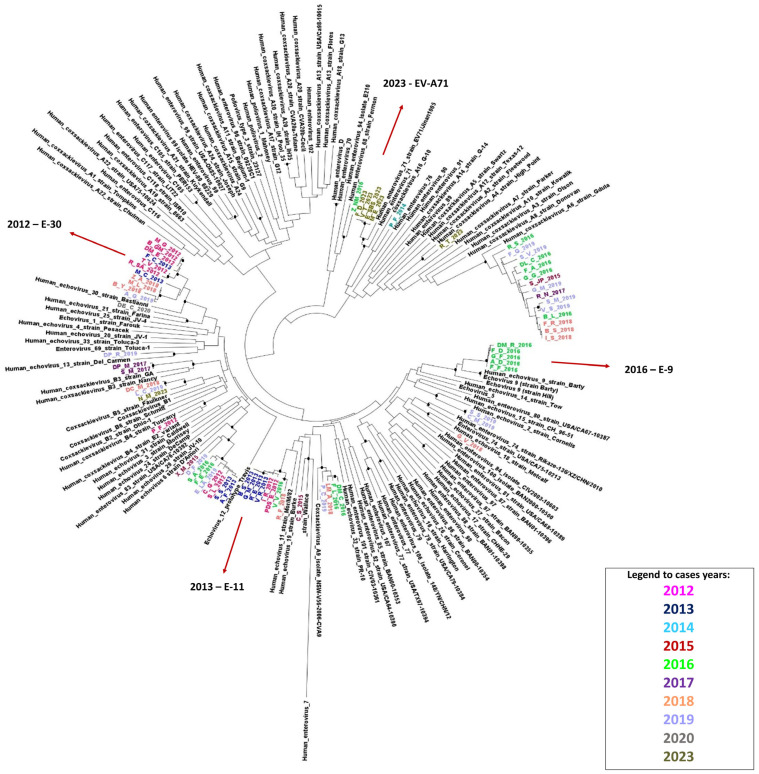
Phylogenetic tree constructed on the basis of partial VP1 sequences of INMI strains circulating in the Lazio region from 2012 to 2023 compared with prototype reference sequences of human EVs of A, B, C and D species. The sequences of INMI strains are indicated by a code and the year of identification; each year is highlighted with a color (as stated in legend of the figure). Red arrows indicate epidemic events and causing types.

Phylogenetic analysis (Figure 5) was performed on the basis of VP1 genes of 70 sequences, respectively, compared to prototype strains of EV types. The topology of the Neighbor Joining tree confirms the data obtained from typing analysis: the tree in Figure 4 clearly shows that each EV type of our patients’ sequences segregates into the corresponding prototype clades, with a significant bootstrap value (>90).

Moreover, observing year distribution of different types, a pattern of EV circulation characterized by both small epidemic outbreaks and sporadic infection is observed. In particular, from the observation of the phylogenetic tree of VP1, isolates of E-6, E-30, CV-A6 and CV-A9 appear to cluster in statistically supported distinct clades (bootstrap > 90) that are, as expected, phylogenetically close to the related prototype sequence, and to diverge into small phylogenetically subclusters closely associated with the year of isolation.

On the other hand, for other types, such as E-11, EV-A71 and E-9, we observed the presence of micro clusters linked to a specific period by phylogenetic analysis, leading to the hypothesis of an epidemic character of infections.

Some other types proved to cause sporadic infections in the geographical area of the Lazio region during the 10 years covered by this study.

## 4. Discussion

In this study a molecular and phylogenetic approach was adopted to study the epidemiology of EVs circulating in the Lazio region by examining the patients referred to the INMI for diagnosis from 2012 to 2023.

During the examined period, 184 cases tested positive for EV infection, mostly distributed uniformly over the analyzed years, with a prevalence ranging from 2% to 11%. Notably, the number of requests for diagnosis strongly decreased in the three years 2020–2022, while they dramatically increased in 2023 with an augmented number of positive EVs cases (11%).

The reduction in the number of performed tests and diagnosed cases could probably be due to the SARS-CoV-2 pandemic that globally affected the three-year period from 2020–2022. During the pandemic period, in fact, the majority of diagnostic efforts were focused on performing tests for SARS-CoV-2. In addition, social distancing measures and the use of personal protective equipment could have contributed to containing the circulation and transmission of other respiratory and fecal–oral viruses, such as EVs.

Whereas the pharmaceutical interventions (e.g., vaccination and medical treatment) that were implemented in order to slow down SARS-CoV-2 transmission are known to be disease specific, non-pharmacological interventions—which included social distancing, mask-wearing, hand hygiene, stay-at-home/lockdown orders, travel restrictions and school closures—are demonstrated to suppress and mitigate the transmission of several endemic notifiable infectious diseases, such as measles and seasonal influenza, and possibly lowered also EV transmission [29,30,31].

As a consequence, the very low circulation of EVs due to the pandemic may have resulted in a lowering of immunity against these infections and, once the social distancing measures were lifted (between 2022 and 2023), may have led to an increased number of EV cases, which we noticed since the end of 2022.

Stratifying the distribution of EVs infection in trimesters, we noticed a seasonal trend of EV infections, with greater peaks in the summer–fall period, according to previous epidemiological studies carried out on a larger scale in the temperate regions of the world [32]. Again, the circulation of EV has changed since the three years affected by the pandemic: at the end of 2020, the coldest period of the year (fall–winter) was affected by EV infection, with major peaks observed in the first and last trimesters of 2023.

For approximately 70% of cases, the EV genome, particularly the 5UTR and/or VP1 partial regions, were sequenced and typed to define species and/or genotype, respectively.

In the period examined by the study, the most frequently encountered species is B, and there is no evidence of circulation of the group C types, to which the 3 poliovirus types belong. In detail, for B species, the most circulating types in the studied period are represented by Echovirus 30 (E-30), Echovirus 6 (E-6), Echovirus 9 (E-9) and Echovirus 11 (E-11).

As regards species A (accounting for 31% of infections), infection with Coxsackievirus A6 (CV-A6) is the most represented followed by EV-A71. For species D, only one case of EV-D68 was diagnosed, which caused a severe infection with neurological symptoms in a highly immunosuppressed woman, as described in the work of Giombini et al. [29]. This type, although it has long been associated with mild infections, has been gaining great attention from the scientific community in the last 10 years. Since emerging in 2014, it has caused a large epidemic of severe respiratory infections in North America, associated with forms of acute flaccid paralysis, similar to that caused by poliovirus [33]. Since then, the virus is still spreading in Europe and North America, with increasing cases.

Moreover, the phylogenetic analysis based on the VP1 region, which has broad information power and resolution capacity, allows some reflections relating to the circulation of the EVs in the Lazio region.

Some types were found in different years of the studied period (as represented by the phylogenetic tree in Figure 4), such as E-6, E-30, E-11, CV-A6 and CV-A9; each of them segregates into single clusters with a significant bootstrap value (>90), and microevolution is observed by the identification of small sub-clusters, closely defined by years of diagnosis. This data displays a genetic drift of the viruses which, in some cases, results in a micro-epidemic circulation pattern of strains. However, further analyses are needed to better define evolutionary dynamics of such strains’ circulation.

As regards the sporadic types, results show that they caused infections distributed over the years, with one or two cases of Coxsackievirus B4 (in 2012), Coxsackievirus A16 (in 2014), Echovirus 7 (in 2015), Coxsackievirus A9 (in 2016), Echovirus 18 (2018), Echovirus 13 (2019), Echovirus 21 (2020) and Coxsackievirus A4 (2023).

Such a situation reflects what can be deduced from the literature regarding the circulation of EV types: the predominant types change over time and, in a given period, suddenly disappear to be replaced by a new one that previously circulated in a less evident way. This characteristic trend is favored by the great genetic variability that characterizes EVs and allows their adaptation to the host and host immunity [11].

Finally, epidemic events that occurred in the Lazio region during the studied period could be observed by the phylogenetic tree: sequences of E-30 in 2012 segregate in a statistically supported well-defined cluster (bootstrap value >90); the same situation occurs for the E-11 sequences of 2013, deriving from an episode of nosocomial epidemic related to a very serious form of hepatitis and myocarditis, the source of which was ascertained with the aid of molecular investigations. In this case, the phylogenetic data obtained from the analysis of the sequences was crucial for revealing the recombinant nature of such a strain [34,35]. Moreover, the E-9 sequences typed in 2016 are also closed together in a single cluster; the same epidemic feature was observed for EV-A71 in 2023, which was found to be recently associated with a nosocomial pediatric epidemic event in the Lazio region.

What was observed in this study suggests that the circulation of E-6, E-30 and CV-A6 is endemic in the Lazio region, while other types are sporadically circulating, sometimes causing outbreaks and epidemic events.

Finally, we could observe a change in circulating types before and after the pandemic three-year period. Despite the short time of observation after the pandemic, there was a noticeable absence of circulation of the previously identified endemic types. Such a condition could be due to prevention measures adopted during the SARS-CoV-2 pandemic, which could consequently have contributed to a change in the immunological status of the population regarding EV infections.

Given the considerations expressed above, we may reach the conclusion that the circulation of EVs is increasing and readapting in the Lazio region. In this scenario, it is crucial to continue to monitor their spread and to improve molecular characterization activities for genomic surveillance purposes.

## 5. Conclusions

In conclusion, the molecular characterization of circulating EV types proved to be of great importance for the identification of the most pathogenic and transmissible EVs variants, and it represents a useful tool in epidemiological surveillance for the purpose of understanding the origin of a specific epidemic outbreak, as well as for associating the onset and spread of a specific type with a peculiar clinical manifestation. Furthermore, the molecular characterization of new or unexpected variants allows us to monitor the emergence of strains that could represent a serious threat to public health if not contained promptly. In particular, optimization of the WGS method could be of great interest in increasing the ability of deeply characterizing strains and thus identifying new emerging recombinant variants at an early stage. For this purpose, a communication network among countries and an integrated system of genomic surveillance could be crucial in allowing for the control of spread and the risk assessment associated with the emergence of new epidemic strains [35]. Moreover, this would also prove to be remarkably important for the study and development of drugs and vaccines, for which significant efforts are being made to find therapies, and prevention measures for those NP-EV types that cause the most serious clinical manifestations [11].

## Data Availability

Human Enterovirus sequences of VP1 and 5′UTR partial region are submitted to NCBI Nucleotide database with the following accession numbers: PP472913—PP473073.

## Supplementary Materials

The following supporting information can be downloaded at: https://www.mdpi.com/article/10.3390/v16071013/s1, File S1: primers and reaction conditions for amplification and sequencing. File S2: dataset of sequences used for phylogenetic analysis.

## References

[1] Pallansch M.A., Roos R.P., Knipe D.M., Howley P.M., Grin D.E., Lamb R.A., Martin M.A., Roizman B., Stephen E.S. (2001). Enteroviruses: Polioviruses, coxsackieviruses, echoviruses, and newer enteroviruses. Fields Virology.

[2] World Health Organization. Regional Office for Europe, Centers for Disease Control and Prevention (U.S.) (2005). Enterovirus Surveillance Guidelines: Guidelines for Enterovirus Surveillance in Support of the Polio Eradication Initiative. https://stacks.cdc.gov/view/cdc/82809.

[3] International Committee on Taxonomy of Viruses Executive Committee (2020). The new scope of virus taxonomy: Partitioning the virosphere into 15 hierarchical ranks. Nat. Microbiol..

[4] Harvala H., Broberg E., Benschop K., Berginc N., Ladhani S., Susi P., Christiansen C., McKenna J., Allen D., Makiello P. (2018). Recommendations for enterovirus diagnostics and characterisation within and beyond Europe. J. Clin. Virol..

[5] Oberste M.S., Maher K., Kilpatrick D.R., Flemister M.R., Brown B.A., Pallansch M.A. (1999). Typing of human enteroviruses by partial sequencing of VP1. J. Clin. Microbiol..

[6] Simmonds P., Gorbalenya A.E., Harvala H., Hovi T., Knowles N.J., Lindberg A.M., Oberste M.S., Palmenberg A.C., Reuter G., Skern T. (2020). Recommendations for the nomenclature of enteroviruses and rhinoviruses. Arch. Virol..

[7] Han Z., Zhang Y., Huang K., Cui H., Hong M., Tang H., Song Y., Yang Q., Zhu S., Yan D. (2018). Genetic characterization and molecular epidemiological analysis of novel enterovirus EV-B80 in China. Emerg. Microbes Infect..

[8] Xiao J., Zhang Y., Hong M., Han Z., Zhang M., Song Y., Yan D., Zhu S., Xu W. (2020). Phylogenetic characteristics and molecular epidemiological analysis of novel enterovirus EV-B83 isolated from Tibet, China. Sci. Rep..

[9] Zhang M., Zhang Y., Hong M., Xiao J., Han Z., Song Y., Zhu S., Yan D., Yang Q., Xu W. (2020). Molecular typing and characterization of a novel genotype of EV-B93 isolated from Tibet, China. PLoS ONE.

[10] Martínez-Salas E. (2008). The impact of RNA structure on picornavirus IRES activity. Trends Microbiol..

[11] Pons-Salort M., Parker E.P.K., Grassly N.C. (2015). The epidemiology of non-polio enteroviruses: Recent advances and outstanding questions. Curr. Opin. Infect. Dis..

[12] Harvala H., Simmonds P. (2009). Human parechoviruses: Biology, epidemiology and clinical significance. J. Clin. Virol..

[13] Jorba J., Campagnoli R., De L., Kew O. (2008). Calibration of Multiple Poliovirus Molecular Clocks Covering an Extended Evolutionary Range. J. Virol..

[14] Ngangas S.T., Lukashev A., Jugie G., Ivanova O., Mansuy J.M., Mengelle C., Izopet J., L’honneur A.S., Rozenberg F., Leyssene D. (2019). Multirecombinant Enterovirus A71 Subgenogroup C1 Isolates Associated with Neurologic Disease, France, 2016–2017. Emerg. Infect. Dis..

[15] Christy A., Messacar K. (2019). Acute Flaccid Myelitis Associated with Enterovirus D68: A Review. J. Child Neurol..

[16] Ma K.C., Winn A., Moline H.L., Scobie H.M., Midgley C.M., Kirking H.L., Adjemian J., Hartnett K.P., Johns D., Jones J.M. (2022). Increase in Acute Respiratory Illnesses Among Children and Adolescents Associated with Rhinoviruses and Enteroviruses, Including Enterovirus D68—United States, July–September 2022. Mmwr. Morb. Mortal. Wkly. Rep..

[17] World Health Organization Disease Outbreak News; Myocarditis (Acute Infective)—United Kingdom. https://www.who.int/emergencies/disease-outbreak-news/item/2023-DON465.

[18] Grapin M., Mirand A., Pinquier D., Basset A., Bendavid M., Bisseux M., Jeannoël M., Kireche B., Kossorotoff M., L’honneur A.-S. (2023). Severe and fatal neonatal infections linked to a new variant of echovirus 11, France, July 2022 to April 2023. Eurosurveillance.

[19] European Centre for Disease Prevention and Control Epidemiological Update: Echovirus 11 Infections in Neonates. https://www.ecdc.europa.eu/en/news-events/epidemiological-update-echovirus-11-infections-neonates.

[20] World Health Organization Disease Outbreak News—Enterovirus Infection—France. https://www.who.int/emergencies/disease-outbreak-news/item/2023-DON469.

[21] Nicholson F., Meetoo G., Aiyar S., Banatvala J.E., Muir P. (1994). Detection of enterovirus RNA in clinical samples by nested polymerase chain reaction for rapid diagnosis of enterovirus infection. J. Virol. Methods.

[22] Nix W.A., Oberste M.S., Pallansch M.A. (2006). Sensitive, seminested PCR amplification of VP1 sequences for direct identification of all enterovirus serotypes from original clinical specimens. J. Clin. Microbiol..

[23] Enterovirus Genotyping Tool.

[24] Saitou N., Nei M. (1987). The neighbor-joining method: A new method for reconstructing phylogenetic trees. Mol. Biol. Evol..

[25] Kumar S., Stecher G., Tamura K. (2016). MEGA7: Molecular Evolutionary Genetics Analysis version 7.0 for bigger da-tasets. Mol. Biol. Evol..

[26] Felsenstein J. (1985). Confidence limits on phylogenies: An approach using the bootstrap. Evolution.

[27] FigTree v. 1.4.4. http://tree.bio.ed.ac.uk/software/figtree/.

[28] Giombini E., Rueca M., Barberi W., Iori A.P., Castilletti C., Scognamiglio P., Vairo F., Ippolito G., Capobianchi M.R., Valli M.B. (2017). Enterovirus D68–Associated Acute Flaccid Myelitis in Immunocompromised Woman, Italy. Emerg. Infect. Dis..

[29] Kuo S.-C., Shih S.-M., Chien L.-H., Hsiung C.A. (2020). Collateral Benefit of COVID-19 Control Measures on Influenza Activity, Taiwan. Emerg. Infect. Dis..

[30] Sullivan S.G., Carlson S., Cheng A.C., Chilver M.B., Dwyer D.E., Irwin M., Kok J., Macartney K., MacLachlan J., Minney-Smith C. (2020). Where has all the influenza gone? The impact of COVID-19 on the circulation of influenza and other respiratory viruses, Australia, March to September 2020. Eurosurveillance.

[31] Nicolay N., Mirinaviciute G., Mollet T., Celentano L.P., Bacci S. (2020). Epidemiology of measles during the COVID-19 pandemic, a description of the surveillance data, 29 EU/EEA countries and the United Kingdom, January to May 2020. Eurosurveillance.

[32] Antona D., Lévêque N., Chomel J.J., Dubrou S., Lévy-Bruhl D., Lina B. (2007). Surveillance of enteroviruses in France, 2000–2004. Eur. J. Clin. Microbiol. Infect. Dis..

[33] Lugo D., Krogstad P. (2016). Enteroviruses in the early 21st century. Curr. Opin. Pediatr..

[34] Rueca M., Lanini S., Giombini E., Messina F., Castilletti C., Ippolito G., Capobianchi M.R., Valli M.B. (2022). Detection of recombinant breakpoint in the genome of human enterovirus E11 strain associated with a fatal nosocomial outbreak. Virol. J..

[35] Fischer T.K., Simmonds P., Harvala H. (2021). The importance of enterovirus surveillance in a post-polio world. Lancet Infect. Dis..

