# Supplementary material for: Molecular Genotyping of Circulating Enterovirus in the Lazio Region from 2012 to 2023
Source: Viruses. 2024 Jun 24;16(7):1013. doi: 10.3390/v16071013 (PMC11281471; doi:10.3390/v16071013)
Supplement: Supplementary file 1 [file viruses-16-01013-s001.zip › viruses-2985006-File S1.pdf]

5UTR primers used for amplification and Sanger Sequencing

EV-U1	GGTGYGAAGAGYCTAYTGAG
EV-D1	CACYGGRTGGCYAATCCA
EV-U2	CCCCTGAATGCGGCTAAT
EV-D2	ATTGTCACCATAAGCAGCCA

EV-U1= I round forward primer; EV-D1= I round reverse primer

EV-U2= II round forward primer; EV-D2= II round reverse primer

[Nicholson et al].

VP1 species-specific primers used for amplification

A Species

I ROUND

VPFAF	TGYRGRTAYTACACNCARTGGTC
VPFAR	GGRTANCCRTCRTARAACCA

VPFAF= forward primer

VPFAR= reverse primer

B Species

I ROUND

	VPFmix A	ATGYTIGGIACICAYDTCATCTGGGA
	VPFmix B	ATGYTIGGIACICAYDTCATATGGGA
	VPFmix C	ATGYTIGGIACICAYDTCATTTGGGA
	VPFmix D	ATGYTIGGIACICAYDTGGTGTGGGA
	VPFmix E	ATGYTIGGIACICAYDTGATTTGGGA
	VPFmix F	ATGYTIGGIACICAYDTGATATGGGA
	VPFmix G	ATGYTIGGIACICAYDTAATATGGGA
VPFmix	VPFmix H	ATGYTIGGIACICAYDTAATTTGGGA
	VPFmix I	ATGYTIGGIACICAYDTAGTTTGGGA
	VPFmix L	ATGYTIGGIACICAYDTAGTATGGGA
	VPFmix M	ATGYTIGGIACICAYDTAGTGTGGGA
	VPFmix N	ATGYTIGGIACICAYDTTGTTTGGGA
	VPFmix O	ATGYTIGGIACICAYDTTGTATGGGA
	VPFmix P	ATGYTIGGIACICAYDTTGTGTGGGA
VPFBR1		TCCCANAYRCARYTNTGCCA
VPFBR2		GGNGCRYTNCCYTCNGTCCA

VPFmix= forward mix of primers

VPFBR1; VPFBR2= reverse primers

D Species

I ROUND

primer F	VPFD68	TGGGACTTTGGATTACAATC
primer R	VPRD68	AGCATTACTGCCTGATTGCCA

VPFD68= forward primer

VPRD68= reverse primer

A, B and D Species

II ROUND

A

AN-89	*CCAGCACTGACAGCA* GYNGARAYNGG
AN-88A	*TACTGGACCACCTGG* GTNGCNGTYTGCCA

AN-89= forward primer [Nix et al].

AN-88B= reverse primer

B

AN-89	*CCAGCACTGACAGCA* GYNGARAYNGG
AN-89B	*CCAGCACTGACAGCA* TGYRTNCCNTGGAT
AN-88	*TACTGGACCACCTGG* NGGNAYRWACAT
AN-88B	*TACTGGACCACCTGG* GTNGANGWTYGCCA

AN-89 [Nix et al]; AN-89B= forward primers

AN-88 [Nix et al]; AN-88B= reverse primers

D

AN-89D	*CCAGCACTGACAGCA* AGAGTGAGAT
AN-89D68	*CCAGCACTGACAGCA* ATGCAGTTGAAACTGG
AN-88D68	*TACTGGACCACCTGG* CTGCCTGATTGCCA

AN-89D; AN-89D68= forward primers

AN-88D68= reverse primer

VP1 - primers used for Sanger Sequencing

AN-232	CCAGCACTGACAGCA
AN-233	TACTGGACCACCTGG

AN-232= forward primer [Nix et al].

AN-233= reverse primer [Nix et al].
